# Dehydrogenation versus deprotonation of disaccharide molecules in vacuum: a thorough theoretical investigation

**DOI:** 10.1098/rsos.220436

**Published:** 2022-10-12

**Authors:** Bohdan Andriyevsky, Nathalie Tarrat, Juan Cortés, Johann Christian Schön

**Affiliations:** ^1^ Faculty of Electronics and Computer Science, Koszalin University of Technology, Śniadeckich Street 2, 74-453 Koszalin, Poland; ^2^ CEMES, Université de Toulouse, CNRS, 29 rue Jeanne Marvig, 31055 Toulouse, France; ^3^ LAAS-CNRS, Université de Toulouse, CNRS, 31400 Toulouse, France; ^4^ Max Planck Institute for Solid State Research, Heisenbergstraße 1, 70569 Stuttgart, Germany

**Keywords:** molecules, sucrose, trehalose, dehydrogenation, deprotonation, density functional theory

## Abstract

Dehydrogenation and deprotonation of sucrose and trehalose molecules in vacuum is theoretically studied by using *ab initio* calculations in the framework of the density functional theory. The differences in the structural, electronic, energetic and vibrational properties of dehydrogenated and deprotonated molecules are discussed, depending on the site from which the hydrogen atom or the proton has been removed. The dehydrogenated molecules are found to be stable, regardless of which hydrogen atom is removed. This contrasts with the instability of the deprotonated molecules, where break-ups or structural reorganizations of the molecule are observed in 20–30% of the cases, but only when the hydrogen atom whose proton is removed was bonded to a carbon atom. Considering the stability and possible rearrangements of the hydrogen network of the deprotonated/dehydrogenated molecule, the formation of additional hydrogen-bridge bonds compared with the nominal molecule appears to be more pronounced for the deprotonated molecules than for the dehydrogenated ones. Moreover, our calculations show that the hydrogen-transfer energy barriers are usually larger for the deprotonated molecules than for the dehydrogenated ones. Finally, compared with the nominal molecule, the vibrational frequency spectrum is shifted to lower frequencies for both the dehydrogenated and the deprotonated molecules.

## 1. Introduction

Usually, the first picture that comes to mind when talking about an organic molecule is the isolated nominal neutral molecule. But there are many situations when a neutral or charged version of the molecule is the more appropriate one, where a hydrogen atom or a proton has been removed, or an electron or a proton has been added. For example, reaction chains often involve neutral radicals [1] and acids easily adopt the deprotonated version of the molecule in solution while bases prefer the addition of a proton. While in these situations additional molecules are present as a solvent or as reaction partners, one also encounters such modified molecules individually in vacuum or the gas phase, e.g. after electrospray ionization (ESI) [2,3], when performing mass spectroscopic experiments on charged molecules [4], or on surfaces after the deposition of single neutral or charged molecules from the gas phase [5–8]. In the latter case, one frequently observes that any excess charge will be neutralized after deposition. This leaves either a dehydrogenated, a nominal neutral or a hydrogenated molecule on the surface, and commonly the hydrogenated molecule will lose its excess hydrogen atom, reverting to the nominal molecule [9].

Among the static and dynamic properties of such isolated molecules, both in vacuum and on surfaces, their feasible conformations including their shape and electronic states, as well as their structural stability, vibrational spectrum and mobility, are of particular interest. Here, the challenge is particularly high for the dehydrogenated and deprotonated molecules, since it is usually not clear which of the hydrogen atoms or protons, respectively, has been removed, and many properties of the molecule, most critically its shape and stability, can be affected by this. Furthermore, not only the overall conformation of the molecule but also the locations of the remaining hydrogen atoms—or equivalently, the location of the hydrogen or proton vacancy in the molecule—can change due to thermally activated processes or proton tunnelling, in principle [10,11]. For both types of processes, the so-called proton internal molecular lability can be estimated from the energy barriers that must be surmounted during such a rearrangement of the hydrogen network [12]. Trying to extract the relevant information solely from experiment is very difficult, especially since the missing atom in question is a hydrogen atom. To address these questions, theoretical analyses are required [13–15].

In this study, we analyse two different disaccharides, sucrose and trehalose, in vacuum. These two molecules have recently been investigated after deposition on metal surfaces as deprotonated modifications via ESI, using scanning tunnelling microscopy methods to study the formation of multi-molecular patterns on the surface, supported by *ab initio* calculations employing the nominal molecules [16,17]. But for a more detailed understanding of the development of these deposits, it is of great importance to understand the state of the molecules and their dehydrogenated and deprotonated modifications in vacuum before they arrive at the surface. Furthermore, the energy landscape, possible shapes and electronic properties of these molecules in vacuum are of general interest, both *sui generis* and as a reference, in addition to serving as a starting point for future simulations of the actual deposition process. Thus, we investigate the nominal, deprotonated and dehydrogenated sucrose and trehalose molecules isolated in vacuum, and determine their properties using density functional theory (DFT) calculations with the VASP code. While the results will only apply directly to the two disaccharides studied, we nevertheless expect that they will also provide insights into the general features of dehydrogenation and deprotonation of disaccharides and more general polysaccharides.

## 2. Models and computational techniques

### 2.1. Models

**Figure 1.**
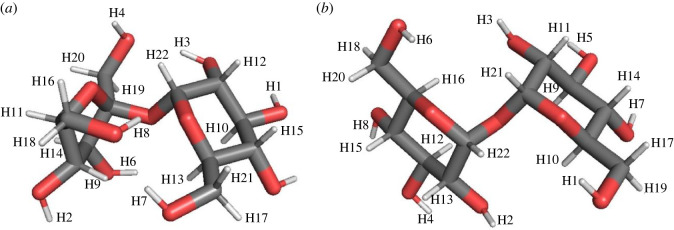
Nominal neutral (*a*) sucrose and (*b*) trehalose molecules. Hydrogen, carbon and oxygen atoms are represented by white, grey and red sticks, respectively.

Sucrose (α-D-glucopyranosyl-(1-2)-α-D-fructofuranoside) and trehalose (α-D-glucopyranosyl-(1-1)-α-D-glucopyranoside) molecules differ only in one of their monosaccharide building blocks (figure 1*a,b*). Trehalose is built of two glucose rings; in sucrose, the first glucose unit is the same as for trehalose, while the second monosaccharide is fructose, in its furanose form (five-membered ring) [18]. To clarify our notation, we call a molecule nominal if it is the original neutral molecule without deprotonation or dehydrogenation having taken place. By contrast, a molecule is called dehydrogenated if one hydrogen atom has been removed and no excess charge is left on the molecule. Finally, a molecule is called deprotonated if it has lost one of its protons and thus looks like a dehydrogenated molecule with an excess charge of one electron.

There are eight H-O bonds and 14 H-C bonds in the sucrose (figure 1*a*) and the trehalose (figure 1*b*) molecules. Depending on the different neighbourhoods of the carbon atoms, one can divide these latter 14 bonds into three subgroups containing seven (H9, H10, H11, H12, H13, H14, H15, called $H_{9-15}^{C(CCO)}$), six (H16, H17, H18, H19, H20, H21, called $H_{16-21}^{C(COH)}$) and one (H22, called $H_{22}^{C(COO)}$) H-C bonds in sucrose, and eight (H9, H10, H11, H12, H13, H14, H15, H16, called $H_{9-16}^{C(CCO)}$), four (H17, H18, H19, H20, called $H_{17-20}^{C(COH)}$) and two (H21, H22, called $H_{21-22}^{C(COO)}$) H-C bonds in trehalose. The numbers in parentheses correspond to the hydrogen labels in figure 1*a*,*b* and in the tables in the electronic supplementary material, Information (SI) file.

To indicate that we are discussing a particular hydrogen atom belonging to the nominal, dehydrogenated or deprotonated molecule of a certain conformation, we first name the conformation, then indicate whether there is a vacancy present. Thus, $s1vH_{1}^{O(C)}$ means that we are dealing with the hydrogen atom no. 1 of the nominal sucrose conformation s1, where the hydrogen atom is bonded to an oxygen atom, which has a bond to a carbon atom. If we are describing a dehydrogenated molecule, then we add a ‘v’ (for vacancy) between the name of the conformation and the hydrogen atom that has been removed, e.g. $s1vH_{1}^{O(C)}$ refers to the sucrose conformation s1 where the hydrogen atom no. 1 has been removed. For the deprotonated molecule, we use P instead of H, to indicate that a proton and not a hydrogen atom has been removed, e.g. $s1vP_{1}^{O(C)}$ refers to the sucrose conformation s1 where the proton no. 1 has been removed. If it is obvious which conformation or modification is presented, the conformation label can be dropped and similarly the ‘v’ label.

Three different conformations were studied for the isolated nominal sucrose (s1, s2 and s3) and nominal trehalose (t1, t2 and t3) molecules in vacuum. These were taken from earlier work investigating the assembly of the nominal disaccharide molecules on a Cu(100) surface [16,17]. These conformations had been obtained with the help of a specially designed robotics-inspired stochastic global optimization method (IGLOO) [19], employing the GLYCAM potential [20], and had been re-optimized using VASP. Both for trehalose and sucrose, the three conformations selected correspond to low-energy minima of the system.

### 2.2. Computational techniques

In a first step, the initial structures of these isolated disaccharides were inserted into a large periodically repeated rectangular box and re-optimized at the DFT level, yielding three sucrose and three trehalose conformations for the neutral nominal molecules. Subsequently, these molecules were deprotonated and dehydrogenated by systematically removing one hydrogen atom / proton from every location of the 22 hydrogen atoms in the molecule (resulting in 22 different singly dehydrogenated /deprotonated molecules for each of the six conformations), followed by another local optimization and an analysis of the resulting dehydrogenated and deprotonated molecules using the Density Derived Electrostatic and Chemical (DDEC6) method [21–24]. The method had been elaborated and carefully tested in the referenced literature [21–24]. Moreover, the charges of the ions calculated by the DDEC6 method were compared to the analogous values obtained using the Gaussian code with the basis set B97D_Def2TZVP containing the polarization orbitals. The corresponding charges for the deprotonated sucrose s1 molecule obtained from these two methods were found to be very close, which can be seen as an indirect proof of the proper use of the DDEC6 method for the molecules studied. Next, intramolecular hydrogen transfer was investigated using the nudged elastic band (NEB) method [25], and, finally, molecular dynamics (MD) simulations of the isolated molecules were performed, allowing the computation of the vibrational spectrum and the radial distribution function (RDF) of the nominal, dehydrogenated and deprotonated molecules.

#### 2.2.1. Energy calculations

The calculations of the electronic properties and the structure minimizations were performed employing DFT, using the VASP code with PAW pseudopotentials [26–31] and the opt-B86b dispersion-corrected functional [32].

A large supercell of about 21.6 × 21.6 × 23.1 Å^3^ was used in order to avoid any spurious interaction between the disaccharides and their periodic images. A cut-off energy for the plane waves of 500 eV, a Methfessel–Paxton smearing *σ* of 0.05 eV [33] and only one irreducible *K*-point, in view of the large supercell, were used for the calculations. The relaxations of the electronic and ionic degrees of freedom were performed with the following default VASP stopping criteria: EDIFF = 1·10^−4^ eV, EDIFFG = −2·10^−2^ eVÅ^−1^. The default criteria for the ending of calculations were used because the structure optimization task performed in the majority of the calculations is a routine procedure of the VASP code and the corresponding characteristic energy differences Δ*E* obtained exceed substantially the default optimization accuracy *δE* ∼ 1 × 10^−4^ eV.

Frequently, a GGA functional is used for describing both structure and energies of solids [34,35], but its local character implies the neglect of the dispersion interactions, which should be considered when studying large molecules and adsorption of molecules on metallic systems [36], as we envision in future work. Thus, in the present calculations, the opt-B86b functional [32] was used, since it has been successfully employed in modelling structural properties of adsorbed molecules with high accuracy. In this functional, a non-local term is added to the local correlation functional to mimic the van der Waals (vdW) London interactions [37]. For the notation of the many different kinds of energies involved in these calculations, we refer to the electronic supplementary material.

#### 2.2.2. Electronic properties

To obtain more information regarding the microscopic characteristics of the objects studied, we used the DDEC6 method described in [21,22]. The DDEC6 method is based on a new theory of bond order (BO) that provides accurate results across an extremely diverse range of materials and bonding types, along with being used to partition the electron and spin densities, and to compute net atomic charges (NACs), dipole moments (DMs), quadrupole moments and BOs. The method allowed us to calculate the overlap population of atom pairs, which may be used to estimate the strength of the hydrogen bonding in a molecule. Similarly, the BO is one of the characteristics that reflects the strength of the chemical bond between two neighbouring atoms. The Chargemol code calculates BOs [23] for every atom in the unit cell on the basis of standard VASP calculations of the charge density. Note that the NACs should be a compact representation of the charge transfer between atoms in materials and also approximately reproduce the electrostatic potential *V*(*r*) surrounding the material; with few exceptions, DDEC6 NACs are similar to the NACs obtained with CHARMM and AMBER [24]. For a consistency check, the charges of the ions calculated by the DDEC6 method were also compared with the analogous values obtained using the Gaussian code [38] with the basis set B97D_Def2TZVP containing the polarization orbitals. Here, a Mulliken population analysis was employed. The corresponding charges for the deprotonated sucrose s1 molecule, obtained by using these two methods, were found to be very close, justifying the use of the DDEC6 method for the molecules studied.

#### 2.2.3. Energy barrier calculations and transition paths

To study the energy barriers encountered during hydrogen migration inside a dehydrogenated or deprotonated molecule in vacuum, we employed the NEB method implemented in the VASP code. With the NEB method, an energy profile along a reaction path is generated using equidistant images along the path. The input geometries of the images are interpolated between the geometries of the initial and final states. Calculations are only done for these intermediate states and the optimization of the geometries is performed under the constraint that the relaxing atoms remain on a plane perpendicular to the (hyper)tangent of the connecting path in configuration space [25,39]. To get a reliable dependence of energy along the NEB path, one should keep in mind that large forces may act on the NEB atom/molecule in some places on the NEB path. Therefore, the corresponding scaling constant for the width of the structure optimization step was chosen to be POTIM = 0.05; this is several times lower than the one used in the case of an ordinary structure optimization. The related threshold value of the forces was chosen to be 0.05 eVÅ^−1^, which is usually sufficiently low to reproduce the corresponding energy barrier and position of the energy maximum on the NEB path with acceptable accuracy.

#### 2.2.4. Molecular dynamics simulations

The above calculations were complemented by MD simulations, which were performed in the canonical NVT ensemble using the VASP code and a Nosé-Hoover thermostat for the temperature (*T* = 40 K; this temperature was chosen as it is a common deposition temperature of such molecules on a substrate [16], which we will analyse in a future publication). Large periodic boxes were used (50 × 50 × 50 Å^3^) to ensure the absence of interactions between the molecule periodic images. Most results were obtained for a simulation time up to 15 ps with time steps of 1.0 fs. The nMoldyn 3.0 [40] program was used for the post-MD analysis. Using the MD-trajectories obtained, the vibrational density of states (VDOS) and the (time averaged) RDF were calculated, with the latter allowing an analysis of the hydrogen bonding in the molecules.

## 3. Results

### 3.1. Structural stability, energetics and charge repartition

#### 3.1.1. Dehydrogenated molecules

We calculate the binding energies of hydrogen atoms at different locations in the sucrose and trehalose molecules. Here, the binding energy of hydrogen atom *i* of molecule *y* (*y* = s: sucrose; *y* = t: trehalose) in vacuum (*v*), ${}_{}^{y}E_{bind}^{Hi;v}$, was evaluated as the difference between the energy of the optimized dehydrogenated molecule plus free hydrogen atom and the energy of the optimized nominal molecule, where the removed hydrogen atom *i* is placed at a distance 10 Å away from the residual (radical) molecule: ${}_{}^{y}E_{bind}^{Hi;v}={}_{}^{y}E_{n;v;r}^{Hi;v;r}+{}_{}^{h}E_{}^{v}-{}_{}^{y}E_{}^{n;v;r}$, where ${}_{}^{y}E_{n;v;r}^{Hi;v;r}$ is the total energy of the relaxed dehydrogenated molecule, ${}_{}^{h}E_{}^{v}$ is the energy of the isolated hydrogen atom and ${}_{}^{y}E_{}^{n;v;r}$ is the total energy of the relaxed nominal molecule. Note that for a molecule, the binding energy ${}_{}^{y}E_{bind}^{Hi;v}$ is a positive quantity by definition since ${}_{}^{y}E_{n;v;r}^{Hi;v;r}+{}_{}^{h}E_{}^{v}>{}_{}^{y}E_{}^{n;v;r}$, while the total energy of a molecule (${}_{}^{y}E_{n;v;r}^{Hi;v;r}$ or ${}_{}^{y}E_{}^{n;v;r}$) is a negative one.

**Figure 2.**
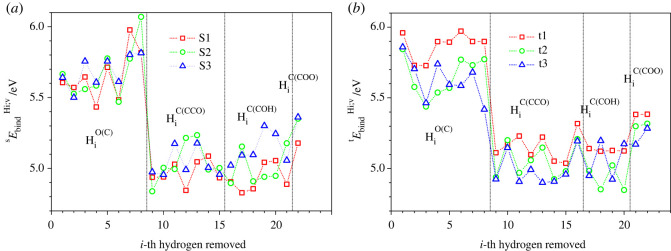
Binding energies of hydrogen atoms, ${}_{}^{y}E_{bind}^{Hi;v}$ (*i* = 1, 2, … ,22), of (*a*) three modifications of sucrose, s1 (red squares), s2 (green circles) and s3 (blue triangles), and (*b*) three modifications of trehalose, t1 (red squares), t2 (green circles) and t3 (blue triangles), as a function of the removed hydrogen atom i. Black vertical dashed-dot lines separate subgroups of the removed hydrogen atoms located near oxygen, $H_{i}^{O(C)}$, and near carbon atoms whose three neighbour atoms are CCO, COH and COO: $H_{i}^{C(\mathrm{CCO})}$ , $H_{i}^{C(\mathrm{COH})}$ and $H_{i}^{C(\mathrm{COO})}$, respectively.

**Figure 3.**
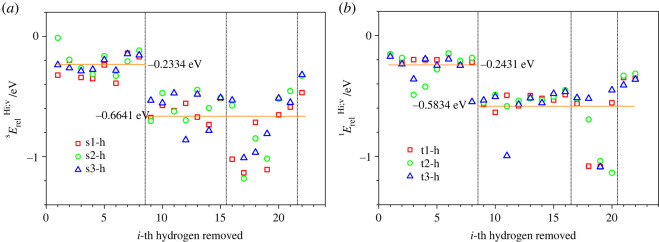
Relaxation energies of dehydrogenated molecules of (*a*) sucrose ${}_{}^{s}E_{rel}^{Hi;v}$ and (*b*) trehalose ${}_{}^{t}E_{rel}^{Hi;v}$ as function of the *i*-th hydrogen atom removed, for which the corresponding specific energies are shown below in figures 5 and 6 for sucrose and trehalose, respectively. In (*a*,*b*), the marker symbols (squares, circles or triangles) correspond to s1, t1 (squares), s2, t2 (circles) and s3, t3 (triangles) modifications of sucrose and trehalose, respectively. The horizontal lines indicate the averaged (over all three conformations and locations where the hydrogen atom has been removed) relaxation energies (shown also in a digital form) of the relaxed dehydrogenated sucrose molecule, where the average is computed separately over those structures where the removed hydrogen atom was located at an oxygen atom (*i* = 1–8) and those where it was located at a carbon atom (*i* = 9–22). The areas bounded by the vertical dashed-dot lines correspond, in the increasing order of *i*-th hydrogen atom removed, to the subgroups $H_{i}^{O(C)}$, $H_{i}^{C(\mathrm{CCO})}$, $H_{i}^{C(\mathrm{COH})}$ and $H_{i}^{C(\mathrm{COO})}$.

**Figure 4.**
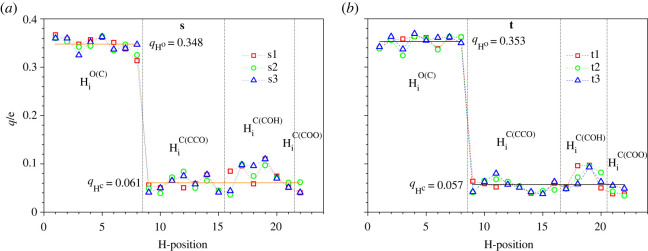
NAC (*q*) of hydrogen atoms for the nominal (*a*) sucrose and (*b*) trehalose molecules. The horizontal lines indicate the averaged NACs over three disaccharide conformations (s1, s2, s3; or t1, t2, t3) and hydrogen atom locations, $H_{i}^{O(C)}$, $H_{i}^{C(\mathrm{CCO})}$, $H_{i}^{C(\mathrm{COH})}$ and $H_{i}^{C(\mathrm{COO})}$.

**Figure 5.**
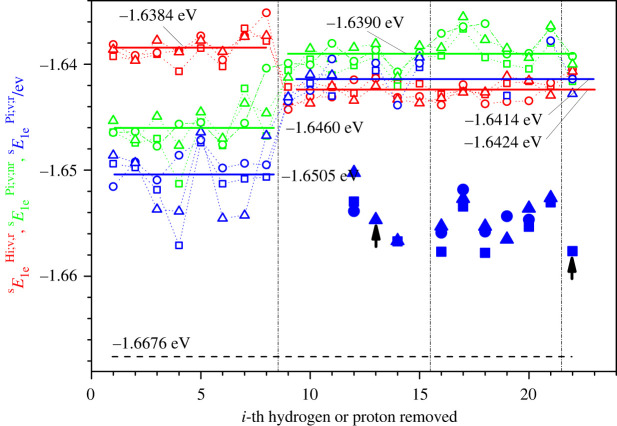
Specific energies ${}_{}^{s}E_{1e}^{Hi,v,r}$, ${}_{}^{s}E_{1e}^{Pi,v,nr}$ and ${}_{}^{s}E_{1e}^{Pi,v,r}$ for sucrose s1 (squares), s2 (circles) and s3 (triangles) as a function of the *i*-th hydrogen or the *i*-th proton removed. Red, green and blue colours correspond to the specific energy of the structurally relaxed dehydrogenated sucrose molecule ${}_{}^{s}E_{1e}^{Hi,v,r}$, the deprotonated molecule after removal of the proton but before structural relaxation $\;{}_{}^{s}E_{1e}^{Pi,v,nr}$ and the deprotonated molecule after structural relaxation $\;{}_{}^{s}E_{1e}^{Pi,v,r}$, respectively. The horizontal lines indicate the averaged (over all three conformations and locations where the proton is removed) specific energies (shown also in a digital form), where the average is computed separately over those structures where the removed proton / hydrogen atom was located at an oxygen atom (*i* = 1–8) and those where it was located at a carbon atom (*i* = 9–22). The filled large markers indicate those cases where the deprotonation leads to a structural instability that results in one of three outcomes: a division of the molecule into two pieces, the destruction of a 5- or 6-membered ring, or a transfer of the proton vacancy to the nearest hydrogen atom bonded to the neighbouring oxygen atom (marked with black arrows). Note that these unstable cases are not included in the energy averages. For comparison, we indicate the specific energy of the nominal sucrose by a black horizontal dashed line.

**Figure 6.**
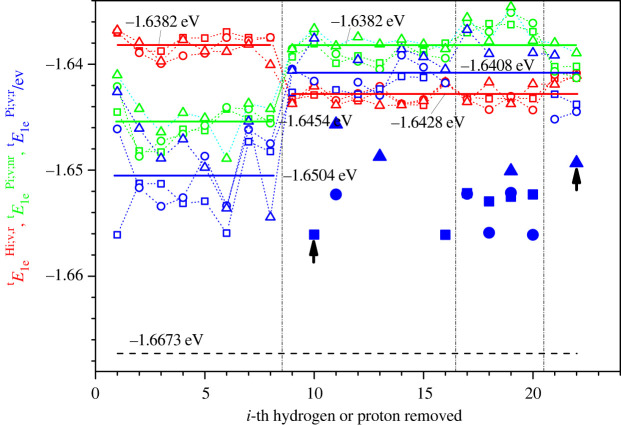
Specific energies ${}_{}^{t}E_{1e}^{Hi,v,r}$, ${}_{}^{t}E_{1e}^{Pi,v,nr}$ and ${}_{}^{t}E_{1e}^{Pi,v,r}$ for trehalose t1 (squares), t2 (circles) and t3 (triangles) as a function of the *i*-th hydrogen or the *i*-th proton removed. Red, green and blue colours correspond to the specific energy of the structurally relaxed dehydrogenated trehalose molecule ${}_{}^{t}E_{1e}^{Hi,v,r}$, the deprotonated molecule after removal of the proton but before structural relaxation ${}_{}^{t}E_{1e}^{Pi,v,nr}$ and the deprotonated molecule after structural relaxation ${}_{}^{t}E_{1e}^{Pi,v,r}$, respectively. The horizontal lines indicate the averaged (over all three conformations and locations where the proton is removed) specific energies (shown also in a digital form), where the average is computed separately over those structures where the removed proton / hydrogen atom was located at an oxygen atom (*i* = 1–8) and those where it was located at a carbon atom (*i* = 9–22). The filled large markers indicate those cases where the deprotonation leads to a structural instability that results in one of three outcomes: a division of the molecule into two pieces, the destruction of a 5- or 6-membered, ring or a transfer of the proton vacancy to the nearest hydrogen atom bonded to the neighbouring oxygen atom (marked with black arrows). Note that these unstable cases are not included in the energy averages. The removed protons with the numbers *i* in the ranges 1–8, 9–16, 17–20 and 21–22 belong to the subgroups $H_{i}^{O(C)}$^)^, $H_{i}^{C(\mathrm{CCO})}$, $H_{i}^{C(\mathrm{COH})}$ and $H_{i}^{C(\mathrm{COO})}$, respectively. For comparison, we indicate the specific energy of the nominal trehalose by a black horizontal dashed line.

**Figure 7.**
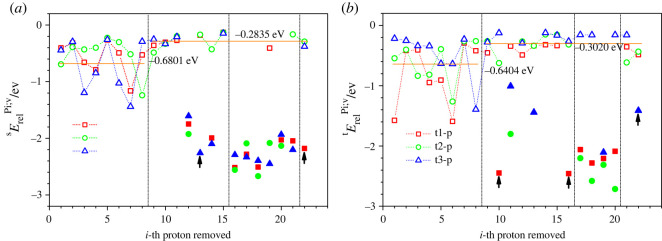
Relaxation energies of deprotonated molecules of (*a*) sucrose ${}_{}^{s}E_{rel}^{Pi;v}$ and (*b*) trehalose ${}_{}^{t}E_{rel}^{Pi;v}$ as a function of the *i*-th proton removed, for which the corresponding specific energies were shown in figures 5 and 6 for sucrose and trehalose, respectively. In (*a*,*b*), the marker symbols correspond to s1, t1 (squares), s2, t2 (circles) and s3, t3 (triangles) modifications of sucrose and trehalose, respectively. The horizontal lines indicate the averaged (over all three conformations and locations where the proton is removed) relaxation energies (shown also in a digital form) of the relaxed deprotonated sucrose molecule, where the average is computed separately over those structures where the removed proton was located at an oxygen atom (*i* = 1–8) and those where it was located at a carbon atom (*i* = 9–22). The filled markers indicate those cases where the deprotonation leads to a structural instability: a split of the molecule into two pieces, the destruction of a 5- or 6-membered ring, or a transfer of the proton vacancy of the nearest hydrogen atom bonded to the neighbouring oxygen atom (marked with black arrows). Note that these unstable cases are not included in the energy averages. For sucrose (*a*), the conformations that are unstable after deprotonation release more energy during this process than the relaxation of deprotonated molecules without structural instability. Overall, the same is true for trehalose (*b*), with just a couple of exceptions (proton 1 and 6 for t1, proton 6 for t2, and proton 8 for t3).

Tables with all the energies of the dehydrogenated and deprotonated molecules—both for relaxed and non-relaxed configurations—are provided in the electronic supplementary material. Note that in the figures 2–7, the subgroups $H_{i}^{O(C)}$, $H_{i}^{C(\mathrm{CCO})}$, $H_{i}^{C(\mathrm{COH})}$ and $H_{i}^{C(\mathrm{COO})}$ encompass the removed hydrogen atoms with numbers *i* in the ranges 1–8 for both sucrose and trehalose, 9–15 for sucrose and 9–16 for trehalose, 16–21 for sucrose and 17–20 for trehalose, and finally 22 for sucrose and 21–22 for trehalose, respectively.

Since there are two types of binding partners for the hydrogen atoms in the disaccharides, i.e. oxygen and carbon atoms, one can expect to find at least two different ranges of binding energies ${}_{}^{y}E_{bind}^{Hi;v}$ of the dehydrogenated disaccharides. This expectation was generally confirmed by the results of the calculations, where the binding energy ${}_{}^{y}E_{bind}^{Hi;v}$ lies between 4.8 eV and 6.0 eV, a variation of about 20% (figure 2). Unsurprisingly, the binding energies are higher for hydrogen atoms bonded to oxygen atoms than for hydrogen atoms bonded to carbon atoms (figure 2). We note that the binding energies vary depending on the conformation of the disaccharide molecule; however, this influence of the molecule's shape is of minor importance compared with the location from which the hydrogen atom / proton had been removed.

Figure 3 shows the relaxation energies ${}_{}^{y}E_{rel}^{Hi;v}$ of the three isomers of sucrose and trehalose after dehydrogenation. Here, a relaxation energy is defined as the energy difference between the non-relaxed and the relaxed molecule after the removal of a hydrogen atom, ${}_{}^{y}E_{rel}^{Hi;v}$, or of a proton, ${}_{}^{y}E_{rel}^{Pi;v}$. Thus, ${}_{}^{y}E_{rel}^{Hi;v}$ is the energy difference between the non-relaxed and the relaxed molecule *y* after removing hydrogen atom *i*: ${}_{}^{y}E_{rel}^{Hi;v}={}_{}^{y}E_{n;v;r}^{Hi;v;r}-{}_{}^{y}E_{n;v;r}^{Hi;v;nr}$; analogously, ${}_{}^{y}E_{rel}^{Pi;v}$ is the energy difference between the non-relaxed and the relaxed molecule *y* after removing proton *i*, ${}_{}^{y}E_{rel}^{Pi;v}={}_{}^{y}E_{n;v;r}^{Pi;v;r}-{}_{}^{y}E_{n;v;r}^{Pi;v;nr}$. For more details about the concept of relaxation energies in the context of molecules, see the electronic supplementary material.

The absolute value of the relaxation energy ${}_{}^{y}E_{rel}^{Hi;v}$ is always lower when the hydrogen atom has been removed from an oxygen atom instead of a carbon one (figure 3). Furthermore, for both disaccharides, the absolute value of the relaxation energy ${}_{}^{y}E_{rel}^{Hi;v}$ upon dehydrogenation is in some cases substantially higher for the second subgroup of carbon atoms C(COH) than those for the other two subgroups C(CCO) and C(COO) (figure 3). This high relaxation energy is caused by the substantial reorientation of the second hydrogen atom bonded to the same carbon atom of the group C(COH) as the removed hydrogen atom. The observed high absolute value of the relaxation energy is a consequence of the re-hybridization of the electronic states near this carbon atom.

Similar differences, depending on whether the hydrogen atoms are bonded to oxygen or carbon atoms, are observed for the NACs (figure 4). This similarity is not accidental because it takes place in all three sucrose and three trehalose modifications. This suggests a close relationship between the relaxation energy mentioned and the NACs of the corresponding hydrogen atoms: the higher the NAC, the lower the absolute value of relaxation energy ${}_{}^{y}E_{rel}^{Hi;v}$ and the less substantial the re-hybridization of the corresponding electronic states. The relaxation energy associated with the dehydrogenation of a molecule ${}_{}^{y}E_{rel}^{Hi;v}$ depends on the atom (oxygen or carbon) from which the hydrogen is removed; in general, the absolute values of the relaxation energies are higher when a hydrogen atom is removed from a carbon atom than when it is removed from an oxygen atom (figure 3). Due to the greater electronegativity of oxygen (*X*_O_ = 3.5) in comparison to carbon (*X*_C_ = 2.5), the electron of an oxygen atom, bonded initially to a hydrogen one before its dehydrogenation (O-H), should be attracted to this oxygen atom more strongly than the analogous electron is attracted to a carbon atom in the case of the dehydrogenation of the C-H bond. This is in agreement with the higher binding energies ${}_{}^{y}E_{bind}^{Hi;v}$ of the hydrogen atoms bonded to oxygen atoms than of those bonded to carbon atoms (figure 2). In turn, lower binding energies ${}_{}^{y}E_{bind}^{Hi;v}$ (positive values) of the hydrogen atoms bonded to carbon atoms result in the higher total energy of the dehydrogenated disaccharides. The latter feature indicates a larger degree of structural instability of the dehydrogenated molecule, which may also lead to the greater instability of the corresponding deprotonated one.

#### 3.1.2. Deprotonated molecules

Next, we turn to deprotonation, where only the proton P_i_ is removed but the electron of the hydrogen atom H_i_ remains (see figures 5–7). To compare the electron bonding in a meaningful fashion in the dehydrogenated and deprotonated molecules, one may use the specific electron energy, i.e. the energy per valence electron (see the corresponding formulae in the captions of figures 5 and 6), because the dehydrogenated and deprotonated molecules have different numbers of electrons (135 and 136, respectively). In particular, we consider the specific energy of the nominal molecule, ${}_{}^{y}E_{1e}^{n;v;r}=\;{}_{}^{y}E_{}^{n;v;r}/N_{e}$ (*N_e_* = 136), of the relaxed dehydrogenated molecule, ${}_{}^{y}E_{1e}^{Hi,v,r}={}_{}^{y}E_{n;v;r}^{Hi;v;r}/N_{e}$ (*N_e_* = 135), of the deprotonated molecule before structural relaxation, ${}_{}^{y}E_{1e}^{Pi,v,nr}={}_{}^{y}E_{n,v,r}^{Pi,v,nr}/N_{e}$ (*N_e_* = 136), and of the deprotonated molecule after relaxation, ${}_{}^{y}E_{1e}^{Pi,v,r}={}_{}^{y}E_{n,v,r}^{Pi,v,r}/N_{e}$(*N_e_* = 136).

As expected, the nominal molecules of the disaccharides have a lower specific energy ($\langle {}_{}^{s}E_{1e}^{n;v;r}\rangle =-1.6676\;\mathrm{eV}$, $\langle {}_{}^{t}E_{1e}^{n;v;r}\rangle =-1.6673\;\mathrm{eV}$) than the relaxed deprotonated ($\langle {}_{}^{s}E_{1e}^{Pi;v;r}\rangle =-1.6487\;\mathrm{eV}$, $\langle {}_{}^{t}E_{1e}^{Pi;v;r}\rangle =-1.6469\;\mathrm{eV}$) and relaxed dehydrogenated ones ($\langle {}_{}^{s}E_{1e}^{Hi;v;r}\rangle =-1.6418\;\mathrm{eV}$, $\langle {}_{}^{t}E_{1e}^{Hi;v;r}\rangle =$ −1.6411 eV); here, $\left\langle {}_{}^{s}E_{1e}^{Pi;v;r}\right\rangle$, $\left\langle {}_{}^{t}E_{1e}^{Pi;v;r}\right\rangle$, $\left\langle {}_{}^{s}E_{1e}^{Hi;v;r}\right\rangle$ and $\left\langle {}_{}^{t}E_{1e}^{Hi;v;r}\right\rangle$ are the averages over all conformations and protons / hydrogen atoms removed. Furthermore, the averaged value $\left\langle {}_{}^{s}E_{1e}^{Pi;v;r}\right\rangle$ is lower for the deprotonated sucrose than for the dehydrogenated one, $\left\langle {}_{}^{s}E_{1e}^{Hi;v;r}\right\rangle$ (figure 5), and the same holds true for trehalose (figure 6).

However, when removing protons, in several cases (20–30%) the atomic configurations become unstable and change substantially by splitting into two separate parts or by a change in the location of the proton vacancy in the sucrose molecules (figures 5 and 7*a*). Similar features are observed for the deprotonated trehalose modifications (figures 6 and 7*b*). Clearly, removing the proton from these sites results in a state with a high energy in a labile configuration, such that the closest local minimum corresponds to a split molecule or involves a reorganization of the hydrogen arrangement.

When removing the proton from an oxygen atom, the specific energies of both the relaxed and the non-relaxed deprotonated molecules are lower than those of the relaxed dehydrogenated molecules (on average). By contrast, the energies of both the non-relaxed and the stable relaxed deprotonated molecules are higher than those of the relaxed dehydrogenated molecules (on average) when we remove the proton from a carbon atom (figures 5 and 6). Furthermore, note that this is the opposite behaviour to the case of the stable dehydrogenated molecules when comparing the specific energy for the case of the removed hydrogen atom being located at a carbon atom and at an oxygen atom (figures 5 and 6).

When the molecule's integrity is preserved after its deprotonation, the absolute value of the averaged relaxation energy ${}_{OH}^{s1}E_{rel}^{Pi;v}$, corresponding to the protons having been removed from an oxygen atom, is higher than the analogous value ${}_{CH}^{s1}E_{rel}^{Pi;v}$, corresponding to the protons having been removed from a carbon atom (figure 7). This result agrees with the greater electronegativity of oxygen compared with carbon: the electron left over after removing the proton from the OH group should be strongly bound to the oxygen atom in comparison to the electron left over when the proton has been removed from a CH group. As a result, the final energies of the deprotonated molecules where the protons had been removed from the positions $H_{i}^{O(C)}$ are lower than those corresponding to the protons having been removed from the positions $H_{i}^{C(\mathrm{CCO})}$, $H_{i}^{C(\mathrm{COH})}$ and $H_{i}^{C(\mathrm{COO})}$. This, in turn, means that the most probable configurations of deprotonated molecules are those with the hydrogen vacancies at the oxygen atoms. This outcome is the opposite to the case of the dehydrogenated molecules (see figures 3, 5 and 6).

In this context, it is of interest to know, for a given molecule and conformation, which hydrogen atom / proton has to be removed in order to yield the lowest energy of the dehydrogenated and deprotonated molecule, respectively, without fragmentation or reorganization. These data are provided in table 1. Note that the location of the hydrogen atom / proton that had been removed in these stable lowest energy structures is different for all the different conformations of sucrose or trehalose. Furthermore, if we also permit fragmentation during the deprotonation of the molecules, then the lowest energy state of the system corresponds to two separate fragments in four of the cases: for all three sucrose conformations s1, s2, s3, and for the trehalose conformation t2.

**Table 1.** The lowest specific energies (in eV) of the stable optimized dehydrogenated and deprotonated conformations of sucrose (s1, s2, s3) and trehalose (t1, t2, t3). In addition, we show in brackets those protons which result in the lowest energy of the system but whose removal leads to the break-up of the corresponding molecules.

molecule conformation	hydrogen atom removed	specific energy of dehydrogenated conformation	proton removed	specific energy of deprotonated conformation
s1	$H_{17}^{C(\mathrm{COH})}$	−1.6461	$P_{4}^{O(C)}$ ; ($P_{18}^{C(\mathrm{COH})}$)	−1.6571 ; (−1.6578)
s2	$H_{9}^{C(\mathrm{CCO})}$	−1.6443	$P_{1}^{O(C)}$ ; ($P_{16}^{C(\mathrm{COH})}$)	−1.6515 ; (−1.6559)
s3	$H_{10}^{C(\mathrm{CCO})}$	−1.6437	$P_{6}^{O(C)}$ ; ($P_{14}^{C(\mathrm{CCO})}$)	−1.6545 ; (−1.6567)
t1	$H_{15}^{C(\mathrm{CCO})}$	−1.6439	$P_{1}^{O(C)}$	−1.6561
t2	$H_{20}^{C(\mathrm{COH})}$	−1.6443	$P_{3}^{O(C)}$ ; ($P_{20}^{C(\mathrm{COH})}$)	−1.6534 ; (−1.6561)
t3	$H_{13}^{C(\mathrm{CCO})}$	−1.6439	$P_{8}^{O(C)}$	−1.6544

If the deprotonation occurs at a carbon atom, then stable disaccharide structures are obtained in approximately half of the cases. For example, in the deprotonated sucrose s1, substantial changes of its structure have been observed in 7 of 14 cases. Such major structural adjustments happen when the protons are removed from C(COH) (five cases), C(CCO) (two cases) and C(COO) (one case) carbon atoms (figures 5 and 7*a*). Similar break-ups or reorganizations were observed during deprotonation of the trehalose t1 molecule. This occurred when deprotonating four C(COH) and two C(CCO) carbon atoms (figures 6 and 7*b*). Typically, the corresponding instability of the deprotonated sucrose/trehalose ion leads to the destruction of the initial disaccharide and the instability is more pronounced when the deprotonation occurs on a C(COH) atom. One should note, however, that this does not mathematically prove that there is not a local minimum for an intact deprotonated molecule—one just cannot find it by simple minimization from the non-relaxed atom configuration after the proton has been removed from the nominal molecule.

Overall, we have found that in quite a number of cases of sucrose and trehalose deprotonation, the molecules become unstable and major re-organizations occur, such as a break-up into two pieces, the opening of a five- or six-membered ring, or the movement of the proton vacancy from its initial location to a new one in the molecule. These results are not completely unexpected, if one takes into account that by deprotonation of the hydrogen atom bonded to a carbon atom, highly unstable species (carbanions) are created (in organic chemistry, carbanions are frequent reaction intermediates [41]). Finally, in some cases, no molecular break-up is observed because local rehybridization is sufficient to stabilize the carbanion.

#### 3.1.3. Electronic charges

More insight into the issue of stability can be gained by considering, e.g., the electronic charges, DMs and BOs in the various molecules. Therefore, we have calculated the atomic charges of sucrose (s1, s2 and s3) and trehalose (t1, t2 and t3) molecules for the nominal, dehydrogenated and deprotonated modifications using the DDEC6 method.

**Figure 8.**
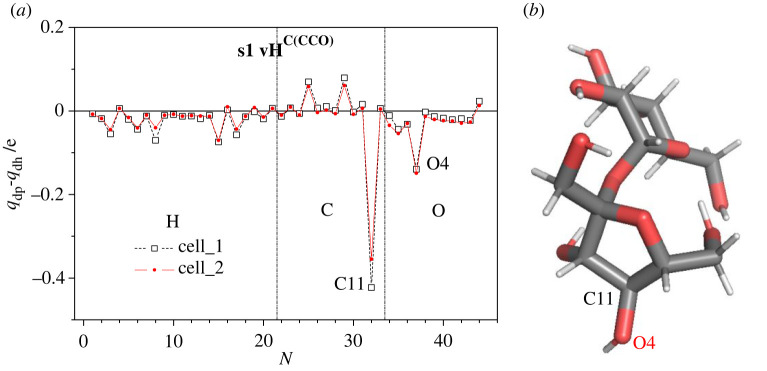
(*a*) Difference in the charge distributions—on an atom-by-atom level—,*q*_dp_ - *q*_dh_, for hydrogen, carbon and oxygen atoms of the deprotonated sucrose s1 compared with the dehydrogenated one. We employed two different simulation cells for these calculations—cell_1 (*V*_1_ = 10.7 nm^3^, *a* = 22 Å) and cell_2 (*V*_2_ = 125 nm^3^, *a* = 50 Å)—, in order to check the possible influence of the cell size on the outcome of the calculations; we note that there are no substantial differences in the charge distributions obtained for two simulation cells of different dimensions. (*b*) View of the atomic structure of dehydrogenated sucrose s1, where the hydrogen atom of the type H^C(CCO)^ has been removed from the carbon atom C11.

As mentioned earlier, the dehydrogenation of the disaccharide molecules does not lead to a breaking of bonds and no substantial changes of their initial atomic structures occur. By contrast, this is not always the case for the deprotonated sucrose and trehalose. To analyse this in more detail, we compare the atomic distributions of the negative charge in the deprotonated sucrose s1 molecule (*q*_net_ = −1 e) with the corresponding dehydrogenated one (*q*_net_ = 0 e) on an atom-by-atom basis (figure 8*a*). We find that the negative excess charge *q*_net_ of the deprotonated molecule is located mainly at the carbon atom (C11) from which the proton had been removed and the neighbouring oxygen atom (O4) indicated in figure 8*b*.

For further analysis of charge and BO aspects of the molecules, we refer to the electronic supplementary material.

### 3.2. Hydrogen transfer energies within nominal, dehydrogenated and deprotonated molecules isolated in vacuum

As we have seen above, an important avenue for structural relaxations and reorganizations is the movement of hydrogen atoms within the molecule. Thus, on the basis of the optimized structures obtained for the nominal, dehydrogenated and deprotonated sucrose and trehalose molecules, the energy barriers for the transfer of hydrogen atoms were calculated using the NEB method [25,39] for a number of illustrative examples.

#### 3.2.1. Nominal molecules

**Figure 9.**
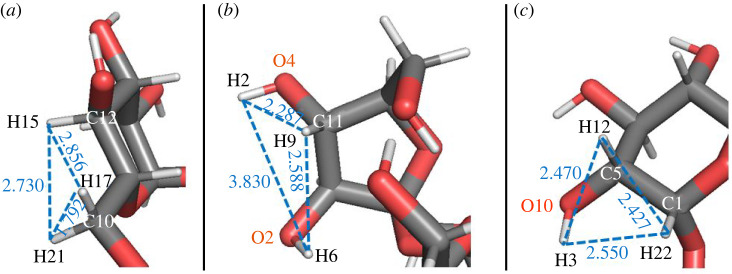
Closed paths of three neighbouring hydrogen atoms in sucrose s1 corresponding to the first three rows of table 2. The interatomic distances are indicated in Angstrom.

In the nominal isolated molecules in vacuum, movement of hydrogen atoms is possible—corresponding to fluctuations in the hydrogen atom locations—, but will always involve the concerted motion of several atoms since transferring only one hydrogen atom would produce an excess hydrogen atom at a carbon or oxygen atom, resulting in unstable and energetically highly unlikely configurations. We calculated the energy barriers for the concerted movement of selected ensembles of three hydrogen atoms that are located close to each other, where we considered five different ensembles for sucrose s1, three for sucrose s2 and four for trehalose t1. For the same number of carbon atoms in a ring (consisting of five or six carbon atoms), three different sets of hydrogen atoms were selected, belonging to different neighbour combinations (H^C(CCO)^, H^C(COH)^ and H^C(COO)^). The specific choices of the three ensembles for the five-membered ring of sucrose s1 are presented in figure 9. The calculated energy barriers for the concerted movement along closed paths of three neighbouring hydrogen atoms are presented in table 2 for sucrose s1 and s2, and in table 3 for trehalose t1.

**Table 2.** Distances, in Å, and energy barriers, in eV, ${}_{NEB;3H}^{s1}E_{n;v;r}^{n;v;Emax}$ for the concerted movement along closed paths of three neighbouring hydrogen atoms H_i_, H_j_, H_k_ of sucrose s1and s2 (called ensembles).

Ens.	closed path of three hydrogen atoms	sum of interatomic distances	NEB path distance	${}_{NEB;3H}^{s1}E_{n;v;r}^{n;v;Emax}$
s1A	H_15_ → H_21_ → H_17_ ⇒ H^C(CCO)^ − H^C(COH)^ − H^C(COH)^ at 6 C-ring	7.38	17.83	3.97
s1B	H_9_ → H_2_ → H_6_ ⇒ H^C(CCO)^ − H^O(C^ − H^O(C)^ at 5 C-ring	8.70	14.27	3.78
s1C	H_22_ → H_3_ → H_12_ ⇒ H^C(CCO)^ − H^O(C)^ − H^C(CCO)^ at 6 C-ring	7.44	8.119	3.20
s1D	H_14_ → H_20_ → H_19_ ⇒ H^C(CCO)^ − H^C(COH)^ − H^C(COH)^ at 5 C-ring	7.02	26.85	3.28
s1E	H_16_ → H_18_ → H_11_ ⇒ H^C(COH)^ − H^C(COH)^ − H^C(CCO)^ at 5 C-ring	6.71	22.0	0.37
s2A	H_19_ → H_20_ → H_14_ ⇒ H^C(COH)^ − H^C(COH)^ − H^C(CCO)^ at 5 C-ring	6.98	13.5	0.007
s2B	H_1_ → H_10_ → H_3_ ⇒ H^O(C)^ − H^C(CCO)^ − H^O(C)^ at 6 C-ring	8.05	41.2	4.46
s2C	H_9_ → H_16_ → H_18_ ⇒ H^C(CCO)^ − H^C(COH)^ − H^C(COH)^ at 5 C-ring	6.76	12.6	0.51

**Table 3.** Distances, in Å, and energy barriers, in eV, ${}_{NEB;3H}^{t1}E_{n;v;r}^{n;v;Emax}$ for the concerted movement along closed paths of three neighbouring hydrogen atoms H_i_, H_j_, H_k_ of trehalose t1 (called ensembles).

Ens.	closed path of three hydrogen atoms	sum of interatomic distances	NEB path distance	${}_{NEB;3H}^{t1}E_{n;v;r}^{n;v;Emax}$
t1A	H_13_ → H_2_ → H_22_ ⇒ H^C(CCO)^ − H^O(C)^ − H^C(COO)^	7.47	6.44	0.08
t1B	H_9_ → H_5_ → H_7_ ⇒ H^C(CCO)^ − H^O(C)^ − H^O(C)^	8.42	21.9	3.17
t1C	H_18_ → H_15_ → H_20_ ⇒ H^C(COH)^ − H^C(CCO)^ − H^C(COH)^	7.44	19.8	3.61
t1D	H_11_ → H_21_ → H_3_ ⇒ H^C(CCO)^ − H^C(COO)^ − H^O(C)^	7.48	9.92	3.95

For 8 of the 12 atom ensembles studied, the energy barriers ${}_{NEB;3H}^{y}E_{n;v;r}^{n;v;Emax}$ of the concerted hydrogen transfers in the disaccharides are larger than 3 eV; only in four cases, these energy barriers are rather small, ${}_{NEB;3H}^{y}E_{n;v;r}^{n;v;Emax}\;<0.52\;\mathrm{eV}$ (tables 2 and 3). In sucrose s1, when two of three hydrogen atoms are of the type H^C(COH)^ and these two atoms are bonded to the same carbon atom, the concerted NEB movement is realized with relatively small energy barriers (see the cases s1E, s2A and s2C in table 2). This is caused by the relatively short distances between these hydrogen atoms of the type H^C(COH)^ (*d*_H-H_ < 1.8 Å) in comparison to the other two distances of these atoms to the hydrogen atom of the type H^C(CCO)^ (*d*_H-H_ > 2.2 Å).

The general features of the concerted NEB movement of hydrogen atoms in trehalose t1 are similar to those observed in sucrose molecules: most of the energy barriers are larger than 3 eV (table 3). However, one notices the unusually small energy barrier (0.082 eV) corresponding to the concerted NEB path H_13_ → H_2_ → H_22_. Here, we do not have any hydrogen atoms of the type H^C(COH)^ as part of the NEB path. In this case, the unusual result is that the sum of interatomic distances is larger than the NEB path distance (columns 3 and 4 of table 3)—the NEB path distance is the length of the hydrogen atom's NEB trajectory. This means that the closest neighbours of the hydrogen atoms H_13_, H_2_ and H_22_ (C, O and C, respectively) move in the opposite direction to the movement of these hydrogen atoms during the NEB path. The total distance of such excess movement is approximately equal to the difference of the distances mentioned, 7.47–6.44 = 1.03 Å.

Ensemble t1A is an example of very close energies and low-energy barriers between positions of three hydrogen atoms (H_13_, H_2_ and H_22_) in the nominal trehalose t1 structure. In several other cases, tables 2 and 3 show that the NEB path distance is 3–5 times larger than the sum of the corresponding interatomic distances. This may be caused by the large divergence of the bond directions, e.g. H_A_-O… and H_B_-C… of the initial structure, and the direction H_A_-H_B_ between the corresponding atoms involved in the given concerted NEB. In this case, the initial parts of the NEB paths of the hydrogen atoms H_A_ and H_B_ may coincide with the bond directions H_A_-O… and H_B_-C…, which may cause the relatively large total NEB paths of H_A_ and H_B_ atoms in comparison to the H_A_-H_B_ distance. Similar path length variations are also observed for the dehydrogenated and deprotonated molecules discussed below.

#### 3.2.2. Dehydrogenated and deprotonated molecules

**Figure 10.**
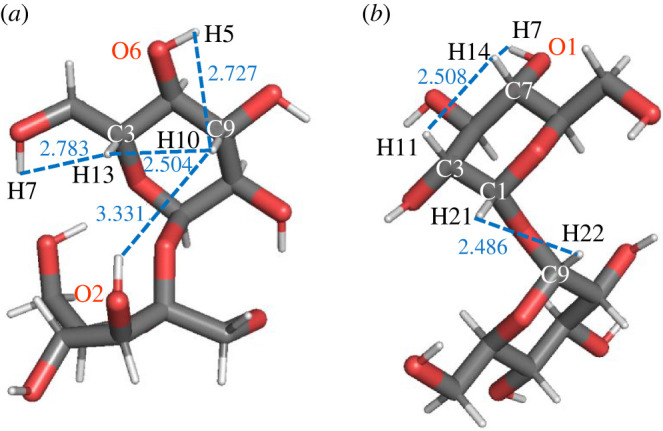
Views of some hydrogen–hydrogen distances in nominal (*a*) sucrose s1 and (*b*) trehalose t1 molecules.

**Figure 11.**
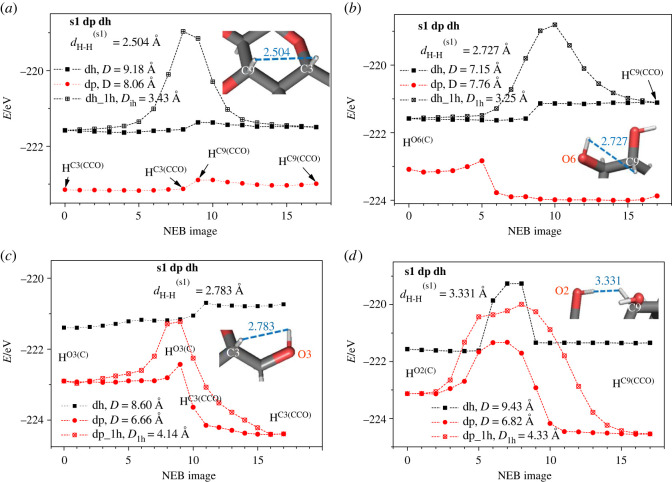
Energy *E* (in eV) versus NEB hydrogen atom images in dehydrogenated (dh) and deprotonated (dp) sucrose s1 for hydrogen transfers between different optimized positions: (*a*) H^C3(CCO)^ − H^C9(CCO)^, (*b*) H^O6(C)^ − H^C9(CCO)^, (*c*) H^O3(C)^ − H^C3(CCO)^ and (*d*) H^O2(C)^ − H^C9(CCO)^. *d*_H-H_ is the interatomic distance H-H between hydrogen atoms that are shifted in the relaxed nominal molecule; *D* is the total distance of the moving ions (*D*_1h_ corresponds to the case when only one hydrogen atom is allowed to be relaxed during the NEB calculation, labelled as dp_1 h or dh_1 h). The nearest carbon and oxygen atoms bonded to the moving hydrogen atom are indicated in the insets.

**Figure 12.**
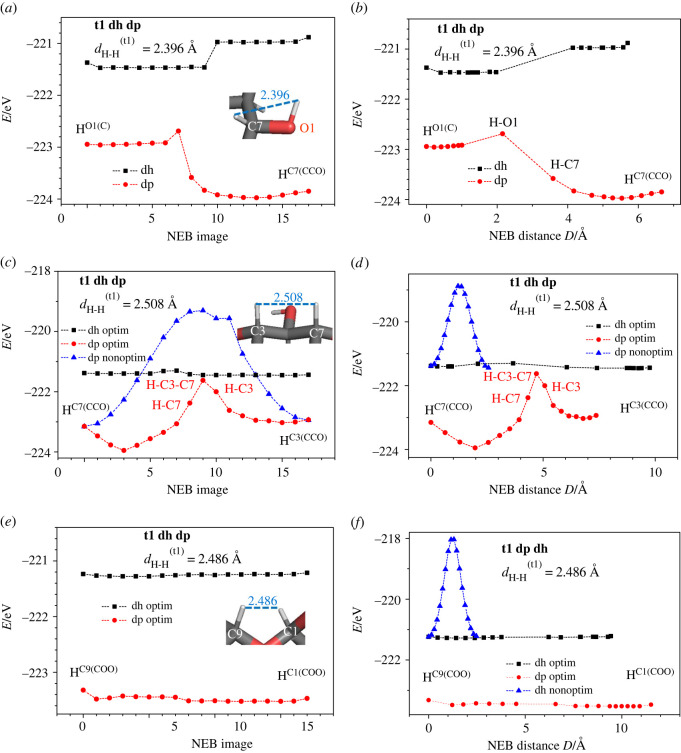
Energy *E* (in eV) versus NEB hydrogen atom images (*a*,*c*,*e*) and versus NEB total distance (*b*,*d*,*f*) in dehydrogenated (dh) and deprotonated (dp) trehalose t1 for hydrogen transfers between different optimized positions: (*a*), (*b*) H^O1(C)^ − H^C7(CCO)^, (*c*), (*d*) H^C7(CCO)^ − H^C3(CCO)^, (*e*), (*f*) H^C9(COO)^ − H^C1(COO)^. *d*_H-H_ is the interatomic distance H-H between hydrogen atoms that are shifted in the NEB calculation in the relaxed nominal molecule; *D* is the total distance of the moving ions. The blue triangles correspond to the NEB states with all atoms fixed besides the moving hydrogen atom or proton. The nearest carbon and oxygen atoms bonded to the moving hydrogen atom are indicated in the insets.

On the basis of the optimized structures obtained for the dehydrogenated disaccharides, calculations of hydrogen migration energy barriers were performed, starting from positions close to the hydrogen vacancies. Here, the goal was to estimate the probability of such migrations of hydrogen atoms, as a function of temperature. For this purpose, the NEB method was used to investigate prototypical transfers (e.g. moving a hydrogen atom located at a carbon atom to a hydrogen vacancy at another carbon or oxygen atom), as presented in figure 10. Note that the hydrogen–hydrogen distances depicted in figure 10*a*,*b* correspond to the optimized nominal disaccharides (with all 22 hydrogen atoms), so they may not exactly equal the real distances between initial and final NEB positions of the dehydrogenated and deprotonated modifications of the molecules. Therefore, the initial and final NEB dehydrogenated/deprotonated structures were optimized before the start of the NEB run; as a result, the spatial positions of all the other atoms apart from the moving hydrogen atom may be a bit different when comparing the initial and final NEB structures. Furthermore, the distance *D* along the actual hydrogen atom NEB trajectory, which includes local relaxations at each NEB step, is, of course, longer than the direct line distance *d* between the initial and final positions of the moving hydrogen atom. The results presented in figures 11 and 12 correspond to the cases when the hydrogen transfers occurred without breaking other interatomic bonds in the sucrose or trehalose molecules.

Quite generally, we note that the energy barriers of seemingly similar NEB paths may be different. This may be illustrated by comparing figures 11*a* and 12*c* for the hydrogen transfers between carbon atoms in the deprotonated sucrose and trehalose molecules. In the case of the higher energy barrier (figure 12*c*), the averaged interatomic distance of the transferring hydrogen atom to the neighbouring atoms is longer than that in the case of figure 11*a*.

Furthermore, the energy barriers are higher when only the transferring hydrogen atom is free to relax (blue triangles in figure 12*c,d,f*), in comparison to the case when all atoms of the molecule are free to move. This is actually to be expected, since a release of constraints can only lead to a lowering of such barriers (unless the removal of the constraints leads to a destruction of the molecule in some fashion—but even then, the barrier would be lower, obviously).

By comparing figure 12*d*,*f*, one may conclude that the larger the energy barrier between the initial and final relaxed hydrogen positions (depicted by blue triangles), the larger the total distance *D* covered by the moving atoms. This is the expected result if one takes into account that the transferring hydrogen atom is exposed to the force field of the neighbouring carbon atoms. Similar effects might be the reason why the energy barrier is almost zero during the hydrogen transfer H^C9(COO)^-H^C1(COO)^ in the vicinity of the bridge oxygen ion of the dehydrogenated and deprotonated trehalose (figure 12*e*,*f*). Note that the distance moved by the hydrogen atom between images varies; furthermore, the distance involved is shortest for the non-relaxed trajectory and longest for the relaxed trajectory of the deprotonated case. For more examples, we refer to the electronic supplementary material.

For dehydrogenated disaccharide molecules, the energy barriers that need to be crossed during hydrogen transfers, ${}_{NEB;1H}^{y}E_{Hi;v;r}^{Hi;v;Emax}$(figures 11 and 12), are found to be smaller than the corresponding binding energies (figure 2), demonstrating that the rearrangement of the hydrogen atoms in the molecule can occur without having to separate the hydrogen atom from the molecule in the process. Another feature is that the energy barriers for hydrogen transfers between hydrogen positions that belong to the same carbon atom are negligible or equal to zero.

In some NEB configurations, the initial and final NEB images of the dehydrogenated molecules are closer in energy than in the cases of the corresponding deprotonated ones (figures 11*b*–*d*, 12*a*; electronic supplementary material, figure SI-12b, SI-12c, SI-12d and SI-13a). This occurs when the hydrogen transfer is realized with the initial and final positions of hydrogen being near oxygen and carbon atoms. The lower energy of NEB transfer is always realized when the hydrogen atom is bonded to a carbon atom and the deprotonated position is near an oxygen atom. The opposite situation (the higher energy of NEB transfer) is observed when the deprotonated position is near a carbon atom. This means that an oxygen atom more strongly accepts/attracts the electron remaining after the deprotonation than a carbon atom. This agrees with the greater absolute value of the electronegativity of oxygen (*X*_O_ = 3.5) compared with that of carbon (*X*_C_ = 2.5). For the deprotonated disaccharides, the energy barriers of hydrogen transfer ${}_{NEB;1H}^{y}E_{Pi;v;r}^{Pi;v;Emax}$ are found to be higher in most cases than those for the dehydrogenated ones ${}_{NEB;1H}^{y}E_{Hi;v;r}^{Hi;v;Emax}$ (electronic supplementary material, figures SI-12 and SI-13).

Since the energy barriers of hydrogen transfer refer to the transfer of one single hydrogen atom without many cooperative effects, the corresponding temperature should be determined from the relation $T={}_{NEB;1H}^{y}E_{Hi;v;r}^{Hi;v;Emax}/k_{B}$. For the energy barriers ${}_{NEB;1H}^{y}E_{Hi;v;r}^{Hi;v;Emax}>2\;\mathrm{eV}$ observed (figures 11 and 12), the corresponding temperatures are too high (*T* > 2.3·10^4^ K) to be achieved in the experiment. We note that even if we assume that, e.g., up to four (next) nearest neighbour atoms at each hydrogen position are strongly involved in this transfer, we still would deal with temperatures in the order of thousands of degrees K. Thus, in these cases, we do not expect hydrogen transfer to be observable in isolated dehydrogenated disaccharides at typical experimental temperatures. However, for some of the nominal (tables 2 and 3) and dehydrogenated (figure 12*e*) molecules, the NEB energy barriers are very small and thus the hydrogen transfer can take place at room temperature.

We note that this situation is different from the one investigated in the study of changes in the hydrogen arrangements of various oligosaccharides [42], where six deprotonated hydrogen atoms belonging to OH groups in one organic molecule were investigated via MD simulation. By contrast, in the current study, only one hydrogen atom or proton is removed from the disaccharide molecule, but for every hydrogen atom in the molecule, the effect of its removal is analysed. As an alternative, one could imagine proton tunnelling processes taking place, but for those paths where the barriers are too high to allow the transfer to take place, e.g. at room temperature, the barrier-distance combination is too large to expect tunnelling to occur instead.

### 3.3. Molecular dynamics of nominal, dehydrogenated and deprotonated sucrose and trehalose molecules

In addition to the NEB investigations, we performed NVT ensemble MD simulations of sucrose and trehalose molecules at *T* = 40 K; this temperature was chosen since it is a typical experimental deposition temperature of molecules on metal surfaces [16]. The goal of this analysis was to investigate the structural (radial and pair distribution functions) and vibrational behaviour (frequencies and amplitude of vibrations), which are expected to affect the deprotonation and dehydrogenation processes of the molecules. In principle, these data can be compared to experimental measurements of the nominal, dehydrogenated and deprotonated conformations of the disaccharide molecules in the gas phase.

**Figure 13.**
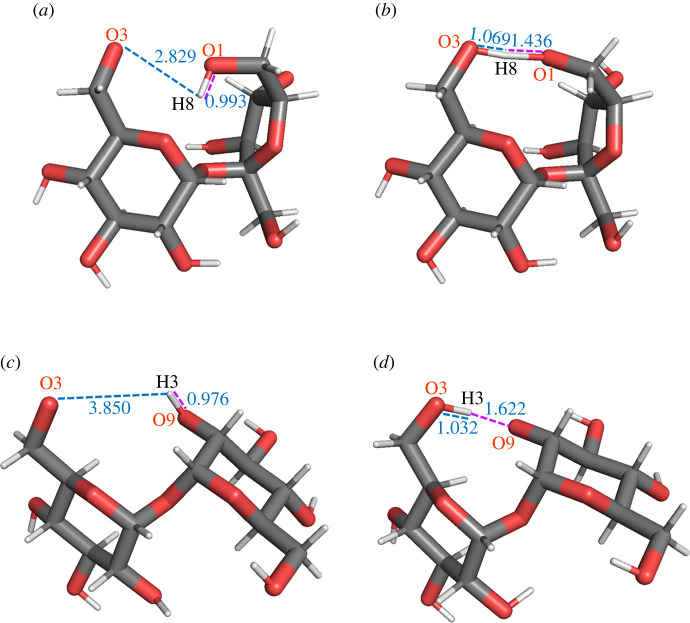
Views of initial, (*a*) and (*c*), and optimized, (*b*) and (*d*), structures of deprotonated s1 and t1 disaccharides, s1vP^O3(C)^ and t1vP^O3(C)^, respectively. The proton was removed from the hydrogen atom, which initially was bonded to the oxygen atom O3: H7 in the case of sucrose and H6 in the case of trehalose.

Before the start of the MD simulation, the atomic structure of the disaccharide molecule studied was locally optimized. For the dehydrogenated and deprotonated molecules, the hydrogen atom / proton was removed from the hydroxyl group O3H. The initial structures of s1 and t1 disaccharides are presented in figure 13, starting with the atom arrangements of the corresponding optimized nominal molecules. During the subsequent structure optimization, a hydrogen atom was transferred from a neighbour atom to the oxygen atom O3: H8 in the case of sucrose and H3 in the case of trehalose.

#### 3.3.1. Atom positions: root mean square deviation and radial (pair) distribution functions

**Figure 14.**
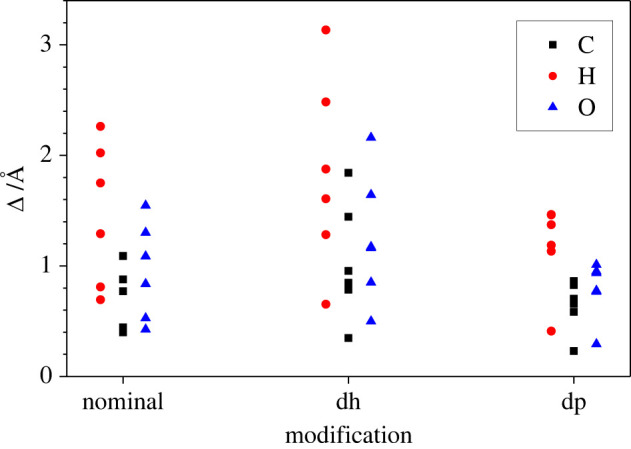
RMSD Δ_H_, Δ_C_ and Δ_O_ of carbon, hydrogen and oxygen atoms for nominal, dehydrogenated (dh) and deprotonated (dp) modifications, respectively, of the three sucrose and three trehalose conformations.

As a measure of the overall structural effects, we analysed the root mean square deviation (RMSD) of carbon, hydrogen and oxygen atoms for the nominal, dehydrogenated and deprotonated versions of the six disaccharide modifications (s1, s2, s3, t1, t2 and t3), where we selected the hydrogen atom that is initially bonded to the oxygen atom O3 in sucrose (figure 13*a*; electronic supplementary material, figure SI-1) and trehalose (figure 13*c*; electronic supplementary material, figure SI-3) as the one to be removed when performing the dehydrogenation/deprotonation. Thus, in total, 18 MD simulations were performed. The values Δ of the RMSD for the particular units (carbon atom, hydrogen atom and oxygen atom) were calculated, and are shown in table 4 and figure 14.

**Table 4.** RMSD Δ_H,C,O_ (in Å) of hydrogen, oxygen and carbon atoms, respectively, in the nominal (nom), dehydrogenated (dh) and deprotonated (dp) sucrose (s1, s2, and s3) and trehalose (t1, t2, and t3) molecules at a temperature of 40 K.

	s1	s2	s3	t1	t2	t3
Δ_H_^(nom)^	2.0231	0.8096	1.2906	0.6947	2.2614	1.7502
Δ_H_^(dh)^	1.8760	3.1349	0.6532	1.2819	2.4834	1.6076
Δ_H_^(dp)^	1.4606	1.1327	0.4101	1.3731	1.1866	1.4642
Δ_O_^(nom)^	1.3009	0.5266	0.8360	0.4245	1.5460	1.0865
Δ_O_^(dh)^	1.1610	2.1600	0.4987	0.8506	1.6427	1.1727
Δ_O_^(dp)^	0.9500	0.7670	0.2932	0.9349	0.7754	1.0101
Δ_C_^(nom)^	1.0900	0.4444	0.7706	0.3978	1.0891	0.8783
Δ_C_^(dh)^	0.9539	1.8420	0.3467	0.7849	1.4439	0.8476
Δ_C_^(dp)^	0.7027	0.5840	0.2304	0.8619	0.6576	0.8262

Figure 14 shows that the relative sizes of the averaged RMSD for the hydrogen, carbon and oxygen atoms, Δ_H_, Δ_C_ and Δ_O_, respectively, are similar for the nominal, dehydrogenated and deprotonated modifications of the disaccharides studied, i.e. we always find Δ_H_ > Δ_O_ > Δ_C_. This is perhaps not too surprising, since carbon atoms are concentrated in the backbone of the molecules, and the hydrogen atoms are much lighter than the oxygen atoms and thus potentially more mobile. However, we also find that the RMSD values Δ_H,C,O_ are lowest for the deprotonated modifications and highest for the dehydrogenated ones (figure 14). We note that this feature correlates with the relationship between the corresponding specific energies of the deprotonated and dehydrogenated molecules (figures 5 and 6).

Besides the overall effect of reducing or increasing the RMSD compared with the nominal molecule due to the deprotonation or dehydrogenation, respectively, it is quite remarkable by how much all the atom-type specific RMSDs vary with the choice of conformation and molecule (s1, s2, s3, t1, t2 and t3).

But in spite of this great variation in the RMSD for the different conformations visible in figure 14, we note some systematics in table 4 when comparing the ratios of the RMSD Δ_H_/Δ_O_ and Δ_H_/Δ_C_, each computed for a given conformation and molecule in the nominal, deprotonated and dehydrogenated modification: the corresponding ratios Δ_H_/Δ_O_ all lie between 1.3 and 1.65, and, similarly, the ratios Δ_H_/Δ_C_ lie between 1.57 and 2.08. This clearly indicates that all modifications of the molecules have a characteristic stiffness: if Δ_H_ is small or large, then Δ_O_ and Δ_C_ are also small or large, and the ratio is quite similar for all modifications. Nevertheless, the relative size of Δ_H_ when considering different molecules and conformations (but the same modification, i.e. nominal, deprotonated and dehydrogenated) can strongly vary when switching from the nominal to the dehydrogenated or deprotonated modification; e.g. $\Delta_{H}^{\left(\mathrm{nom}\right)}(s1)>\Delta_{H}^{\left(\mathrm{nom}\right)}(s2)$, but $\Delta_{H}^{\left(\mathrm{dh}\right)}(s1)<\Delta_{H}^{\left(\mathrm{dh}\right)}(s2)$, while $\Delta_{H}^{\left(\mathrm{dp}\right)}(s1)>\Delta_{H}^{\left(\mathrm{dp}\right)}(s2)$. Finally, we note that the RMSD can be interpreted as a measure of the atom-projected amplitudes of the vibrational modes of the molecule.

**Figure 15.**
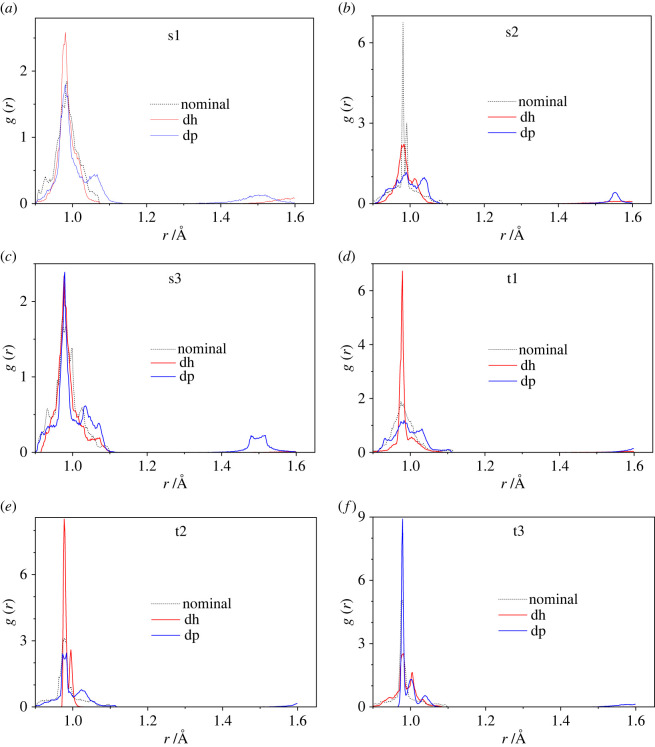
RDF *g*(*r*) of all hydrogen–oxygen atomic pairs for sucrose (*a*) s1, (*b*) s2 and (*c*) s3, and for trehalose (*d*) t1, (*e*) t2 and (*f*) t3, conformations for nominal (dotted black), dehydrogenated (red) and deprotonated (blue) modifications obtained using results of MD at the temperature of 40 K.

In addition, we considered the RDFs of the disaccharides that incorporate information about all interatomic distances C–C, C–H, C–O, H–H, H–O and O–O (electronic supplementary material, figure SI-14). For distances up to 3 Å, these distribution functions look very similar, where the peaks can be assigned to the pairs of neighbouring atoms H–C, H–O, C–C and C–O. In figure 15, we focus on the comparison of the O-H atom pair RDFs for the nominal, dehydrogenated and deprotonated molecules in the range 0.9–1.6 Å, since we are particularly interested in the changes in the O-H bond lengths.

The main maximum at *r* ≍ 0.98 Å is due to the single O-H bonds in molecules. The main consequence of the dehydrogenation and deprotonation of disaccharides is the appearance of additional peaks of the RDF corresponding to the O–H distances *r* in the range 1.0–1.1 Å. The RDF maxima in this range are due to the additional interaction of the hydrogen atom forming the short O-H bonds with the slightly more distant oxygen atom/ion that has an unsaturated bond due to the dehydrogenation or deprotonation. In the three deprotonated sucrose modifications, these external oxygen atoms are located at a distance *r*_H…O_ = 1.5 Å (figure 15*a–c*), so that an additional hydrogen bonding O-H … O interaction becomes relevant in the sucrose molecule due to its deprotonation. In the three trehalose modifications, the additional peaks in the RDF in the range 1.0–1.1 Å also exist, but the analogous maxima of the RDF at *r*_H…O_ = 1.5 Å are less pronounced (figure 15*d–f*). This result indicates a greater ability of the sucrose modifications to create an additional internal hydrogen bonding of the type O-H … O upon dehydrogenation and deprotonation in comparison to the trehalose modifications.

**Figure 16.**
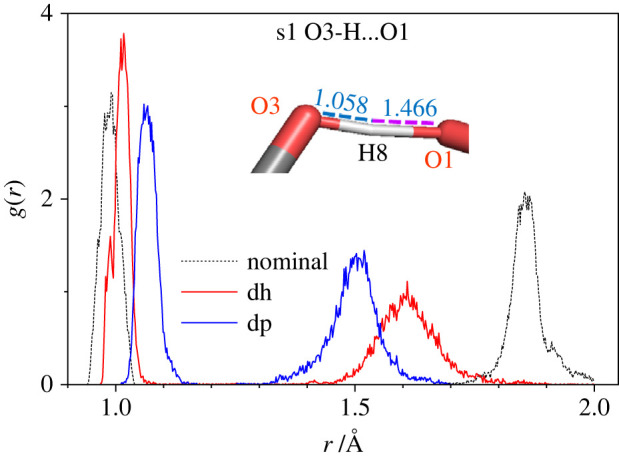
RDF *g*(*r*) of the atoms involved in the hydrogen bonding O3-H8…O1 for nominal (dotted black), dehydrogenated (red) and deprotonated (blue) s1 sucrose modifications. Inset: fragment of deprotonated sucrose s1 atomic structure showing the hydrogen bonding O3-H8…O1.

Additional features due to the creation of additional hydrogen bonds are seen in figure 16, where the RDFs of only the three atoms involved in the hydrogen bonding O3-H8…O1 are presented for the nominal, dehydrogenated and deprotonated modifications of sucrose s1.

This new hydrogen-bridge bond occurs between the two carbon rings of sucrose s1. The shortening of the distance O3-O1 and the increase of the distance O3-H8 are observed when switching from the nominal to the dehydrogenated or the deprotonated sucrose s1 molecule. We note that it may also be possible that due to small overall deformations of the s1 atomic configuration—as might happen at higher temperatures—,the position of the hydrogen atom shown in the inset of figure 16 (H8-O3 is the short half of the hydrogen-bridge bond) may change, such that the H8-O1 bond will become the short part of the hydrogen-bridge bond. This may happen when, e.g., the absolute value of the negative charge on the O1 atom becomes greater than that on the O3 one, and thus the short part of the hydrogen bond O3-H8…O1 switches from O8 to O1, i.e. O3…H8-O1.

Taking into account the classification of hydrogen bonds by Jeffrey [43] and the RDF obtained (figures 15 and 16), one can characterize the hydrogen bonding strength in the disaccharides studied as being on a moderate strong level. Differences of RDFs for sucrose and trehalose in the range of 1.5–1.6 Å (figure 15) are a substantiation of the claim that the hydrogen bonding in the sucrose molecule is a bit stronger than that in the trehalose one. Furthermore, the results presented in figure 16 suggest that the hydrogen bonding O-H…O becomes stronger when moving from the nominal over the dehydrogenated to the deprotonated disaccharides.

#### 3.3.2. Vibrational density of states

**Figure 17.**
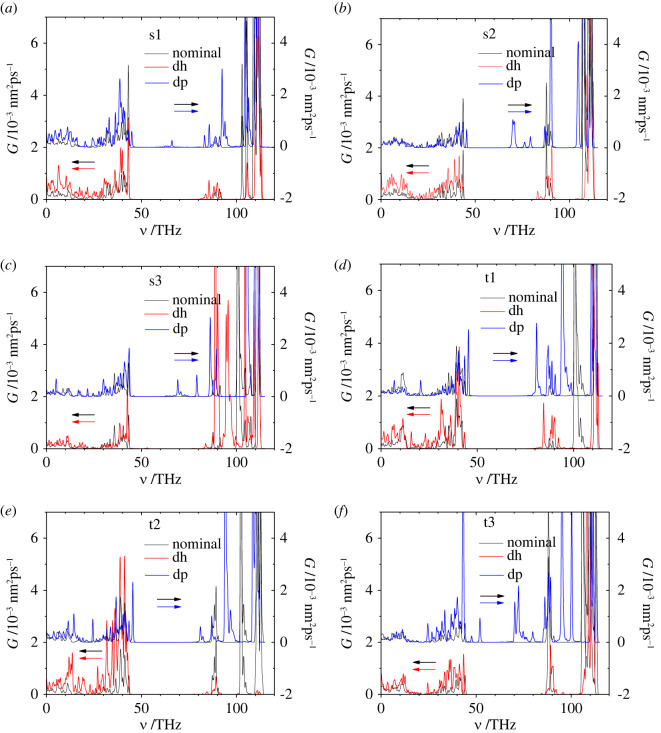
VDOS as function of frequency *ν* for nominal (black), dehydrogenated (red) and deprotonated (blue) sucrose (*a*) s1, (*b*) s2 and (*c*) s3, and trehalose (*d*) t1, (*e*) t2 and (*f*) t3 modifications obtained using results of MD at the temperature of 40 K. In order to compare the VDOS of the nominal molecule separately with the deprotonated and the dehydrogenated molecule, we plot the nominal curve twice, but once shifted upwards by 2 units (note that the *y*-axis to the right is shifted up by two units compared with the *y*-axis on the left). The VDOS of the dehydrogenated molecule refers to the *y*-axis on the left, while the VDOS for the deprotonated molecule is shifted by two units upwards, referring to the *y*-axis on the right. The corresponding *y*-axis assignment is indicated by the red, blue and two black arrows.

The VDOS derived from the MD simulations for the 18 molecules considered are shown in figure 17. For the nominal molecules, the high-frequency range of VDOS (*ν* > 100 THz) mainly involves the hydrogen atoms, while the vibrations in the low-frequency range (*ν* < 45 THz) are mainly associated with the carbon and oxygen atoms. The positions of the frequency maxima in the calculated VDOS of trehalose agree with the results of the corresponding experimental measurements [44–47].

Differences between nominal, dehydrogenated and deprotonated molecules can be seen in the VDOS (figure 17). In most cases, the deprotonation leads to shifts of the VDOS maxima in the high-frequency range of 85–115 THz to somewhat lower frequencies, which indicates the increased hydrogen atom interaction with the neighbouring charged oxygen units, and thus the weakening of the isolated O-H and C-H bonds that are responsible for the high frequencies of the associated localized vibrational modes. Similarly, in most cases, the VDOS peaks of the dehydrogenated sucrose and trehalose molecules are located at lower frequencies than those for the nominal analogues, or the corresponding relative amplitudes of the VDOS peaks in the low-frequency range (*ν* < 50 THz) are higher for the dehydrogenated molecules (figure 17). This is closely related to the observation that additional interaction terms are present in the dehydrogenated and deprotonated molecules compared with the nominal disaccharides, which lead to somewhat stronger intra-molecular forces, in agreement with the approximate correlation (electronic supplementary material, figure SI-11) between the H-O overlap populations *Π*_H-O_—serving as an indicator of the degree of intra-molecular hydrogen-bridge bonding—and the total energy *E*.

**Figure 18.**
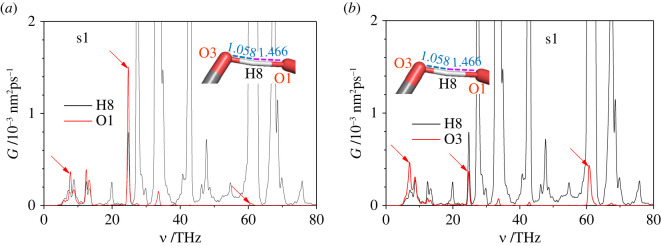
VDOS as function of frequency *ν* of sucrose s1 (*a*) hydrogen atom H8 and oxygen atom O1, and (*b*) hydrogen atom H8 and oxygen atom O3 (only three atoms, H8, O1 and O3, were free to move during the MD).

One of the effects of dehydrogenation and deprotonation is the appearance of new peaks at frequencies that are different from those for the nominal modifications of the disaccharide molecules. This effect is more pronounced for the deprotonated molecules than for the dehydrogenated ones. These new peaks are again caused by the increased interaction of the oxygen and/or carbon atoms with the hydrogen atoms in the dehydrogenated/deprotonated disaccharide molecule in comparison to the nominal one. Such interactions are expected to decrease the vibration frequency of the bonds H-O- or H-C-, especially if the additional action occurs from the opposite side of the oxygen or carbon atoms of these bonds. To verify this hypothesis, we performed a constrained MD simulation of the deprotonated sucrose s1, where only three atoms, O1, H8 and O3, were allowed to move (figure 18).

We find that the VDOS of the hydrogen atom H8 obtained for the nominal and deprotonated sucrose s1 are substantially different. For example, the maximum frequency in the deprotonated sucrose s1 is 60–70% lower than that in the nominal one (figure 17*a*). This result supports the above hypothesis. The comparable features of the VDOS for the oxygen O1 and O3 atoms are in agreement with their distances to the hydrogen atom H8 in between. The VDOS spectrum of the O1 atom is shifted to the low-frequency range in comparison to the similar value of the oxygen O3 atom (figure 18). Also, the high-frequency vibration mode at *ν* = 61 THz is hybridized more for the O3-H8 bond than for the O1-H8 one; this is in qualitative agreement with the interatomic distances of the hydrogen bonding O3-H8…O1 (figure 18).

## 4. Discussion

The effects of dehydrogenation and deprotonation of two disaccharide molecules, sucrose and trehalose, are observed in structural changes relative to the nominal molecules, such as break-ups, deformations and reorganizations of the network of the remaining hydrogen atoms. Furthermore, the vibrational spectrum is modified and the intramolecular bonding, especially via hydrogen bonds, can change.

Starting with the changes in the structure of the molecules, we observe that the dehydrogenated molecules are stable, regardless of which hydrogen atom is removed. This contrasts with the instability of the deprotonated molecules, where break-ups or structural reorganizations of the molecule are observed in 20–30% of the cases, but only when the hydrogen atom whose proton is removed was bonded to a carbon atom. This is probably due to the greater electronegativity *X* of oxygen (*X*_O_ = 3.5) than of carbon (*X*_C_ = 2.5), resulting in a more stable electron configuration of the negative oxygen ion compared with the negative carbon one in all three modifications of sucrose and trehalose. For some of those molecules that did not split into two pieces and where a hydroxyl group is located very close to the hydrogen or proton vacancy, a transfer of the vacancy to the neighbouring oxygen atom can happen with essentially no energy barrier preventing the transfer. In the case of deprotonation, we notice that the remaining excess electron charge stays mostly at the atom—carbon or oxygen—from which the proton is removed. If another oxygen atom is located near such an oxygen or carbon atom, then this oxygen atom also becomes negatively charged, again due to the high electronegativity of oxygen.

From an energetic point of view, we note that directly after the deprotonation of a carbon atom, the molecule contains a large amount of potential energy before it has had chance to relax structurally. In *ca* 50% of these cases, the molecule can relax into a local minimum structure without breaking up or re-organizing itself. In practice, this means that the molecule is still at a high-energy level and thus probably quite susceptible to, e.g., reorganizations of the hydrogen distribution in the molecule, which might be caused, e.g., by fluctuations of the structure at a given temperature. However, if there is no OH group close enough to donate its hydrogen atom without having to cross an energy barrier, then the molecule can sometimes remain in the high-lying local minimum configuration—the local gradient-based minimization effectively takes place at zero temperature. By contrast, in the other 50% of cases, there is a straight downhill path to a break-up of the molecule or to a hydrogen transfer within the molecule without an impeding energy barrier; thus, the molecule follows this route, releasing its stored up potential energy and reaching an energetically more stable state with rather low energy, even though this low-energy state of the system might consist of two separate fragments of the original molecule.

While certain features, such as whether the hydrogen atom or proton is removed from an oxygen atom or carbon atom, are of major importance and show clear trends regardless of the molecule and conformation (s1, s2, s3, t1, t2 and t3) being considered, we also observe noticeable variations in, e.g., the binding and relaxation energies of the dehydrogenated and deprotonated molecules, when comparing for a given molecule (sucrose or trehalose) the three conformations after a particular hydrogen atom or proton has been removed. Thus, the stability and relaxation behaviour of a disaccharide molecule clearly depends on the type of conformation it exhibits during the dehydrogenation or deprotonation process.

Besides the spontaneous hydrogen atom transfers to the location of the hydrogen/proton vacancy that sometimes occur during the relaxation stage after the removal of the hydrogen atom / proton, hydrogen atoms or protons can also move inside the molecule by crossing small energy barriers without involving a complete separation of the hydrogen atom from the molecule. Thus, the energy barriers and hydrogen transfer paths for possible reorganizations of the hydrogen network of the various dehydrogenated and deprotonated molecules have been investigated. For the deprotonated disaccharides, the energy barriers encountered during hydrogen transfer are observed to be higher, in most cases, than those for the dehydrogenated molecules. Furthermore, for the deprotonated sucrose and trehalose molecules, the lengths of the paths taken by the hydrogen atom according to the NEB calculations are found to be substantially longer than those of the analogous atom transfer paths in the dehydrogenated molecules. These features may be due to the repulsive forces between the migrating hydrogen atom / proton and the residual part of the dehydrogenated/deprotonated molecule. Since the energy barriers are higher for the deprotonated disaccharides, one would estimate that a rearrangement of the hydrogen network of an isolated dehydrogenated molecule might be feasible at experimental temperatures, while the deprotonated molecule would most likely be unchanged unless exposed to rather high temperatures. Indeed, in the absence of an environment, there is nothing to stabilize the transient charges that exist during a proton transfer.

One also observes differences between the dehydrogenated and deprotonated molecules when considering the VDOS, where the deprotonation has a greater effect than the dehydrogenation. In both cases, new VDOS maxima (not present in the VDOS of the nominal molecules) appear in the range of 40–85 THz, possibly due to the influence of additional interactions among the atoms, which weaken the original ionic-and-covalent-like H-O and H-C bonds. As a result, the force constants that control the (mostly local) high-frequency vibrations associated with the H-O and H-C bonds are reduced, leading to a decrease in the shift of these frequencies. In particular in the case of those deprotonated molecules where the proton is removed from a hydroxyl group, the hydrogen-bridge bonding O-H…O associated with these hydroxyl groups becomes more relevant; this is reflected in the changes of the RDF at the interatomic distances in the ranges of 0.1–0.11 nm (short hydrogen bonding bond length) and 1.4–2.0 Å (long hydrogen bonding bond length).

## 5. Conclusion

In this study, we have investigated the effects of dehydrogenation and deprotonation on two disaccharides, sucrose and trehalose. We have systematically considered the removal of each of the 22 hydrogen atoms / protons separately, demonstrating quite a large variation in the binding and relaxation energies, charge distributions, and structural rearrangements during the relaxation after removal of the atom or proton—up to break-ups of the molecule for 20–30% of the choices of the removed proton.

The results could be classified according to the local environment of the hydrogen atom concerned; we not only observed the expected difference when removing the hydrogen atom / proton from an oxygen or carbon atom, but also found among the carbon atoms a further grouping of the outcomes, depending on which other atoms were directly bound to this carbon atom. This general classification appeared to hold for all six conformations considered (three for trehalose and three for sucrose), but there was still a noticeable variation both within each group and for different conformations of the molecules. Clearly, the quantitative, and also qualitative, effects of a dehydrogenation or deprotonation depend on both the local bond structure around the atom from which the atom/proton was removed and on the more global shape of the conformation.

Compared with the nominal molecule, the vibrational frequency spectrum is shifted to lower frequencies for the dehydrogenated and deprotonated molecules. Comparing the effects of deprotonation and dehydrogenation, we note that the major difference is that none of the dehydrogenated molecules suffered a break-up in contrast with several of the deprotonated molecules. Furthermore, our NEB studies of the energy barriers and hydrogen atom paths associated with the movement of hydrogen atoms in the dehydrogenated and deprotonated molecules showed that these barriers are usually larger for the deprotonated molecules than for the dehydrogenated ones. Considering the formation of additional hydrogen-bridge bonds compared with the nominal molecule, it appears that this effect is more pronounced for the deprotonated molecules than for the dehydrogenated ones.

Quite generally, we can conclude that removing a hydrogen atom or a proton from the nominal disaccharide molecules can lead to a large variety of outcomes, depending on which hydrogen atom / proton is removed, but also to some degree on the conformation of the disaccharide molecule. While some aspects of the results are in agreement with qualitative or semi-quantitative arguments, the overall variability of the properties of the molecules after removing a hydrogen atom or a proton from different locations on the molecule underlines the importance of systematic theoretical studies of such molecules. This will surely also be true for future investigations of the glycans, or the study of the removal of two or more hydrogen atoms or protons not only from saccharide molecules but also from biomolecules in general.

## Data Availability

The main data are accessible in the paper and in the corresponding Supplementary Information file: https://doi.org/10.5281/zenodo.7155564.
